# Giant Patent Ductus Arteriosus Aneurysm Compressing the Esophagus

**DOI:** 10.3400/avd.avd.cr.23-00047

**Published:** 2023-09-28

**Authors:** Soichiro Henmi, Chikashi Nakai, So Izumi, Yutaka Nakashima, Takuro Tsukube

**Affiliations:** 1 Division of Cardiovascular Surgery, Japanese Red Cross Kobe Hospital, Kobe, Hyogo, Japan; 2 Division of Pathology, Japanese Red Cross Fukuoka Hospital, Fukuoka, Fukuoka, Japan

**Keywords:** patent ductus arteriosus, aneurysm, total arch replacement

## Abstract

It is extremely rare to observe aneurysmal changes in patients with patent ductus arteriosus (PDA), especially in adults. If left untreated, a PDA aneurysm can increase the risk of life-threatening complications, including rupture, dissection, esophageal fistula, and infection. Following is a description of successful surgical repair in a 55-year-old man with PDA aneurysm compressing the esophagus. Histologically, the aneurysmal wall showed mild thickening of the intima and media with hyperplastic smooth muscle cells, but no destructive changes were observed.

## Introduction

Aneurysmal changes in patients with patent ductus arteriosus (PDA) are extremely rare, especially in adults. Most reported cases were found in newborn babies and children aged below 3 months. Most adults with ductus arteriosus aneurysm (DAA) were of non-patent type and often diagnosed either at autopsy or unexpectedly during cardiac surgery.^1,2)^ Following is a description of successful surgical repair in a 55-year-old man with PDA aneurysm compressing the esophagus.

## Case Report

**Figure 1:**
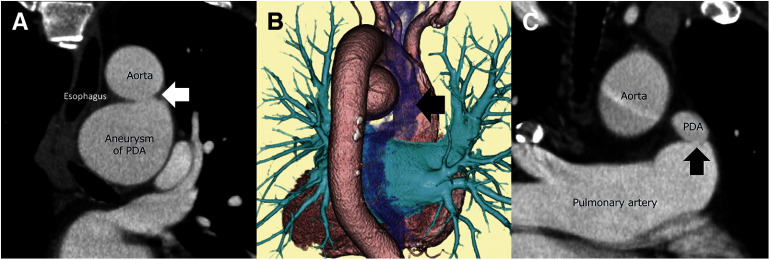
Fig. 1 Preoperative contrast-enhanced CT image. (**A**) PDA aneurysm compressing on the esophagus, and the aortic side orifice (white arrow) with a diameter of 14 mm. (**B**) The aneurysm located under the aortic arch and aneurysmal sac measured 57 × 50 mm. It exerted pressure on the esophagus (black arrow, blue colored: esophagus). (**C**) The pulmonary side orifice (black arrow) with a diameter of just 4 mm. CT: computed tomography; PDA: patent ductus arteriosus.

A 55-year-old man presented with severe dysphagia, and a contrast-enhanced computed tomography (CT) revealed a PDA aneurysm that was compressing the esophagus (**Fig. 1A**). The CT showed that the aneurysm was located under the aortic arch and was in contact with both the aorta and the pulmonary artery (PA). The orifice in the aorta was 14 mm in diameter (**Fig. 1A**); the aneurysmal sac measured 57 × 50 mm, which was compressing the esophagus (**Fig. 1B**); and the PA orifice was 4 mm in diameter (**Fig. 1C**). A cardiac echocardiogram demonstrated the presence of abnormal continuous flow (left to right) in the dilated main PA. Furthermore, the ratio of pulmonary to systemic blood flow was determined to be 1.73. The coronary angiography did not reveal any significant findings.

**Figure 2:**
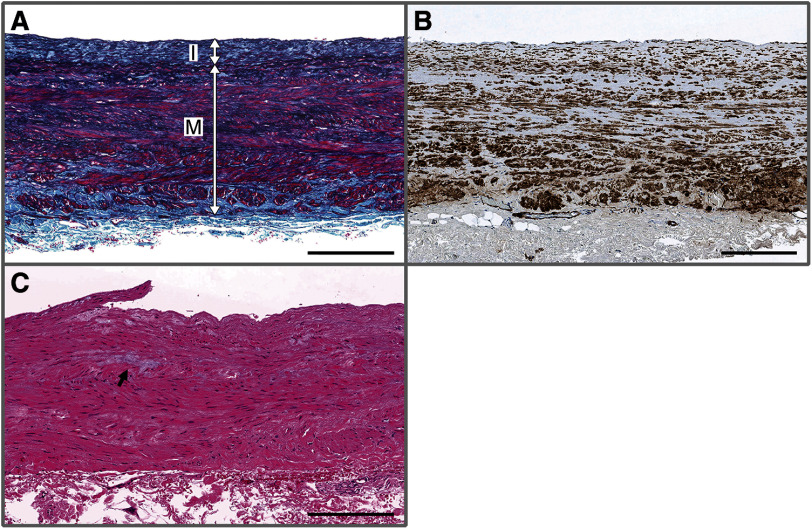
Fig. 2 Histology of aneurysmal wall. (**A**) Elastica-Masson stain, (**B**) immunohistochemistry for α-SMA, and (**C**) H&E stain. The arrow indicates a tiny mass of cystic medial necrosis (**C**). Bars represent 250 μm. SMA: smooth muscle actin; H&E: hematoxylin and eosin; I: intima; M: media.

A total arch replacement was performed via median sternotomy. Under moderate hypothermic circulatory arrest cooling to 29°C at the rectal temperature and with selective antegrade cerebral perfusion, a distal aorta was transected 1 cm distal to the orifice of the PDA. After the distal aortic stump and 22 mm–3 branch Dacron graft (Japan Lifeline Co., Tokyo, Japan) were anastomosed, the orifice between the PDA and PA was directly closed. Circulatory arrest time was 41 minutes and cardiopulmonary bypass time was 143 minutes. During the pathological examination, the aneurysmal wall showed mild thickening of the intima and media without atherosclerotic changes (**Fig. 2**). Smooth muscle cells were densely arranged in the media and appeared to be hyperplastic (**Figs. 2A** and **2B**). A tiny mass of cystic medial necrosis was found in the media (**Fig. 2C**). Otherwise, no significant destructive change was observed. After surgery, the patient no longer complained of dysphagia, hoarseness, or any neurological dysfunction and a postoperative CT confirmed that the aorta had been successfully repaired. The patient was discharged 8 days after the surgery.

## Discussion

The mechanism of DAA formation remains uncertain, but two theories have been proposed to explain its pathogenesis. One is the delayed closure or persistent opening of the aortic end of the ductus arteriosus; the other is the congenital or acquired weakening of the wall.^3)^ While histological examinations are useful to explore the pathogenesis, there are very few case studies demonstrating the histology of the wall of a DAA. Falcone et al. revealed that elastic fibers in the aneurysmal wall were fewer in number than those in the aortic and pulmonary walls.^1)^ Fukuta et al. noticed the fibrous thickening of the intima and disappearance of medial elastic fibers in the aneurysmal wall.^4)^ In our case, hyperplastic changes in the smooth muscle cells appeared in the media of the aneurysmal wall. Based on our findings, Ohtsuka et al. also reported similar observations in their study of an adult case with aneurysm of the PDA. They described focal necrosis and mucoid degeneration of the media, along with intimal thickening and fibroelastosis in the PDA aneurysm. These findings align with our own research and provide additional support to the understanding of the pathological features of PDA aneurysms in an adult case.^5)^ In addition, its anatomical characteristics included an aortic orifice that was larger than the pulmonary orifice. Considered together, these findings suggest that the ductus arteriosus was dilated to form an aneurysm because its wall had been exposed to systemic pressure for a long period.

We performed conventional total arch replacement on the patient, directly closing the PA orifice. Alternatively, patch closure at the aortic and pulmonary end together with resection of the aneurysm or interventional exclusion of the aneurysm would have been one of options. Patch closure in the aortic side orifice might be easy, but it also required circulatory arrest during closure. In addition, there remains the risk of formation of pseudoaneurysm. Thoracic endovascular aortic repair including the frozen elephant trunk technique is considered to be another option. Saito et al. reported the successful endovascular repair of the DAA^6)^ but with a closed pulmonary end of the PDA. In our case, the remaining blood flow on the PA side might promote the expansion of the aneurysmal sac even when the aortic side is closed by a stent graft. Also, we have to take care of postoperative aortoesophageal fistula following endovascular aortic repair even without any endoleak.^7,8)^ In the present case, the patient had already suffered from dysphagia preoperatively so that releasing the pressure from the aneurysmal sac to the esophagus had to be necessary.

## Conclusion

We could operate successful surgical repair to a PDA aneurysm in a 55-year-old man. Total arch replacement and directly closing of the orifice in the PA were alternative procedures in such a case.

## Informed Consent

We have obtained written informed consent from the patient for reporting this case.

## Disclosure Statement

The authors have no conflicts to declare.

## Author Contributions

Study conception: SH and TT

Data collection: SH and YN

Analysis: SH and YN

Investigation: SH

Writing: SH and CN

Funding acquisition: None

Critical review and revision: all authors

Final approval of the article: all authors

Accountability for all aspects of the work: all authors.
